# Supplementary material for: MC profiling: a novel approach to analyze DNA methylation heterogeneity in genome-wide bisulfite sequencing data
Source: NAR Genom Bioinform. 2022 Dec 31;4(4):lqac096. doi: 10.1093/nargab/lqac096 (PMC9803872; doi:10.1093/nargab/lqac096)
Supplement: lqac096_Supplemental_File [file lqac096_supplemental_file.docx]

**Supplementary Figures**

***Supplementary Figure 1: Results from simulated data.* A:** Accuracy of MC profiles from simulated datasets with increased coverage (y-axis: average JSD value of the MC profiles estimated between 1000 low-coverage 4-CpG datasets and the MC profile computed from the high-coverage 4-CpG dataset. x-axis: number of reads to simulate low coverage datasets. Dashed lines: gain in accuracy when increasing the coverage between 25 and 50 reads. Solid lines: interval of increased coverage to obtain the gain in accuracy observed between 25 and 50 reads). **B:** Precision of MC profiles estimated from simulated 50 reads datasets for each 4-CpG high coverage dataset (y-axis: JSD values between MC profiles estimated from 1000 datasets’ pairs; x-axis: 4-CpG datasets).


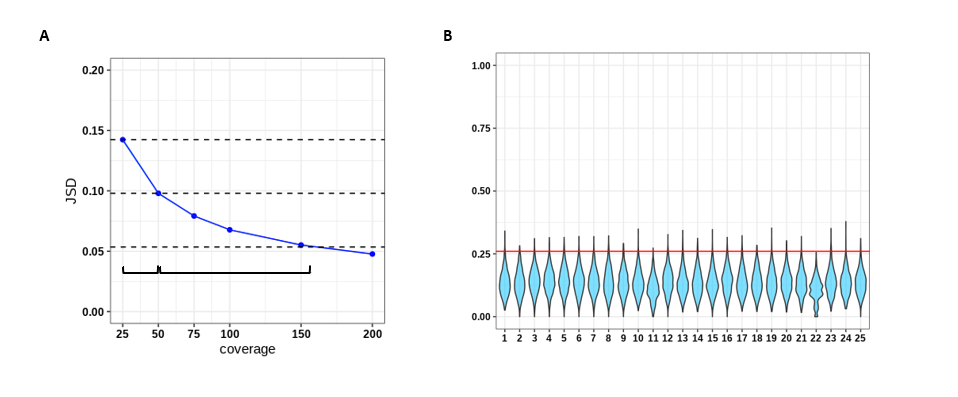


***Supplementary Figure 2: Appropriateness of data compression scheme for Dataset 1 (panel A) and Dataset 2 (panel B).*** For each MC profiles, the within class distance (WCD), i.e. the JSD between the MC profiles to the most similar Methylation Pattern (MP) is indicated on the x-axis, whereas the External Class Distance (ECD), i.e. the JSD from the second most similar MP, is shown on the y-axis. The MC profiles whose ECD/WCD ratio was higher than 1.5 (95% of MC profiles for Dataset 1 and 98% of MC profiles for Dataset 2) are colored in blue.

**
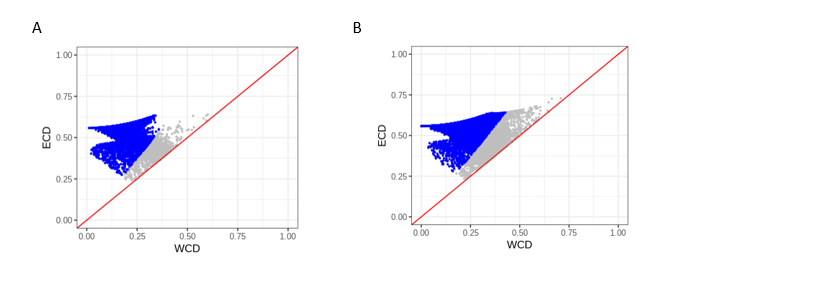
**

**Supplementary Figure 3: Number and genomic distribution of analyzed epiloci. A:** number of epiloci in three samples from Dataset 1 (M1:3) and Dataset 2 (H1:3). B: annotation of epiloci in respect of CpG density and genic regions in three samples from Dataset 1 (M1:3) and Dataset 2 (H1:3).

**
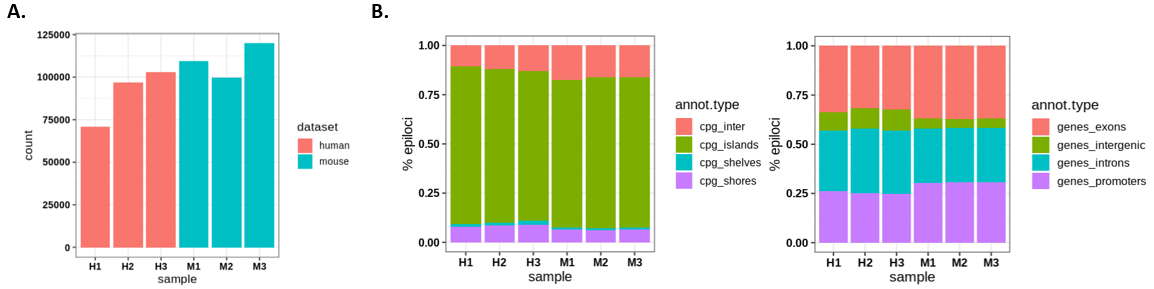
**

**Supplementary Figure 4: Fraction of genomic functional regions holding at least 1 epilocus in Dataset 1 (panel A) and Dataset 2 (panel B) samples**

**
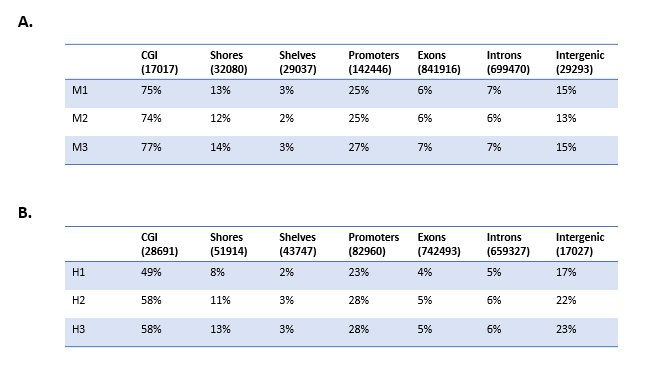
**

**Supplementary Figure 5: Average DNA methylation of epiloci assigned to the different MPs in Dataset 1 (first row) and Dataset 2 (second row) samples**

**
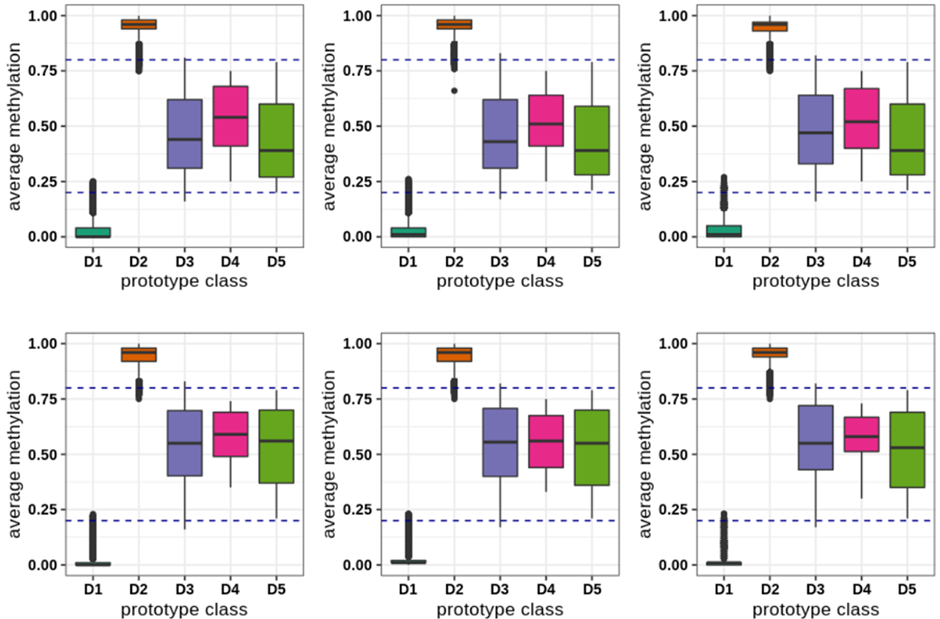
**

***Supplementary Figure 6: MC profiling results for Dataset 2.* A:** Distribution of MC profile distance between sample pairs. The red line indicates the cutoff of JSD. **B:** Genomic annotation of epiloci with stable or variant MC profiles. **C:** average distance between epiloci inside concordant and discordant bins (Wilcoxon test p-value 5.624e-13). **D:** Fraction of epiloci attributed to the different MPs. **E:** genomic annotation of epiloci assigned to the different MPs.

**
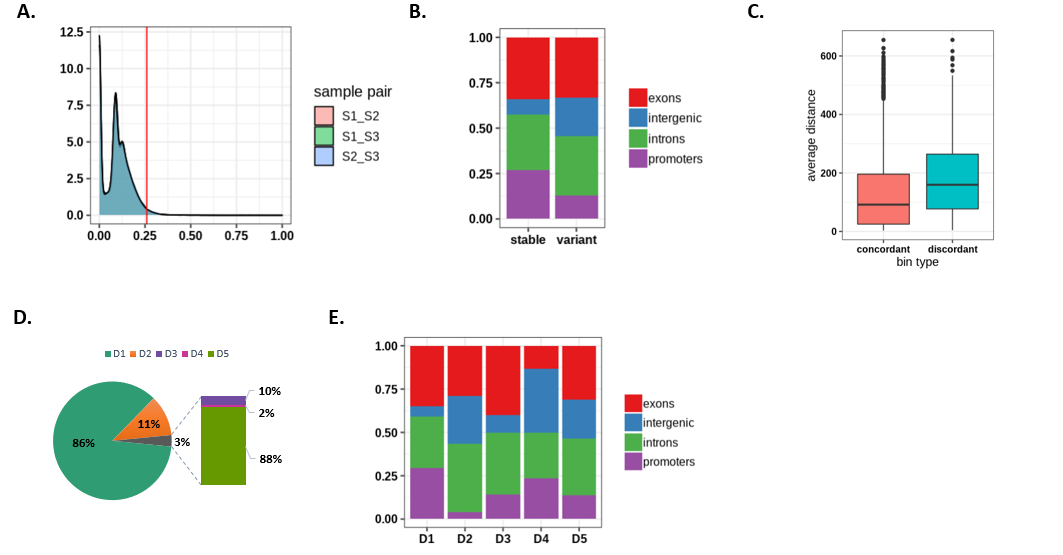
**

**Supplementary Figure 7: Distribution of concordant bin numbers resulting from bootstrapping analysis in Dataset 1(A) and Dataset 2(B).** In both panels, the red line indicates the observed value


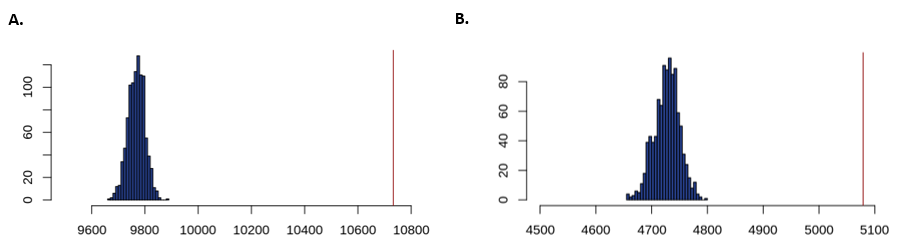


***Supplementary Figure 8: MP composition across functional regions in Dataset2. A.*** *Proportion of MPs for regions assigned to different functional categories according to the chromHMM track for GM12878 cel line. (Tx= Strong Transcription, EnhG= Genic enhancer, Quies= Quiescent/Low, TxWk= Weak transcription, Het= Heterochromatin, TxFlnk= transcription at gene 5’ and 3’, ZNFRpts= ZNF genes and repeats, Enh= enhancer, ReprPCWk= Weak Repressed Polycomb, TSSAFlnk= Flanking active TSS, ReprPC=Repressed Polycomb, EnhBiv= Bivalent enhancer, BivFlnk=Flanking bivalent TSS/enhancer, TssBiv= Bivalent/Poised TSS, TssA= Active TSS).* ***B.*** *Proportion of MPs in functional regions of active genes. C. Proportion of MPs in functional regions of inactive/lowly-expressed genes.*

**
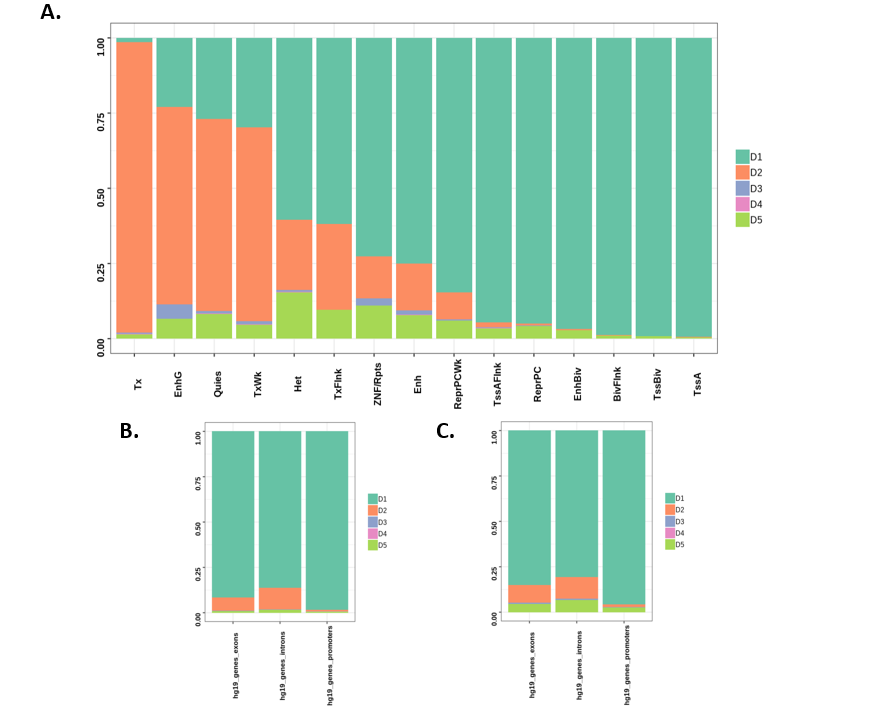
**

**Supplementary Figure 9: Contribution of cellular heterogeneity to MC profiles at polymorphic epiloci.** Molecular heterogeneity of joined MC profiles in three samples from Dataset 3. The y-axis indicates the MC count value, i.e. the number of MCs with non-zero relative abundance. The x-axis indicates the proportion of epiloci with a given MC count.


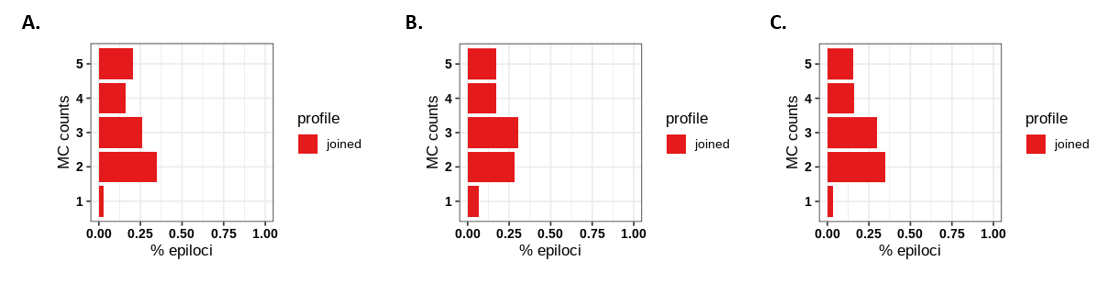


**Supplementary Figure 10: Proportion of epiloci assigned to the different MPs in imprinted and non imprinted genomic regions in the Dataset 2.**


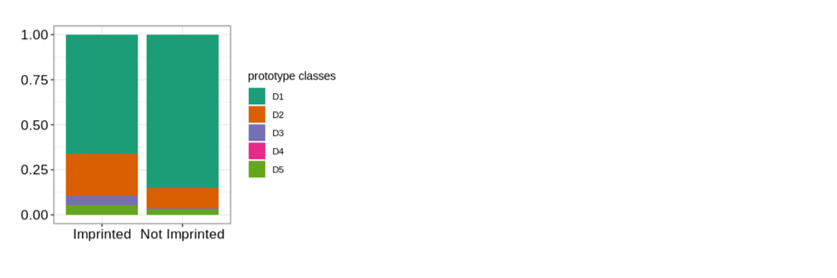


**Supplementary Figure 11: Gene set enrichment analysis for genes associated with 5129 epiloci with changes in MC profiles and stable average methylation upon differentiation.** Genes associated with all analyzed epiloci were used as background.

**
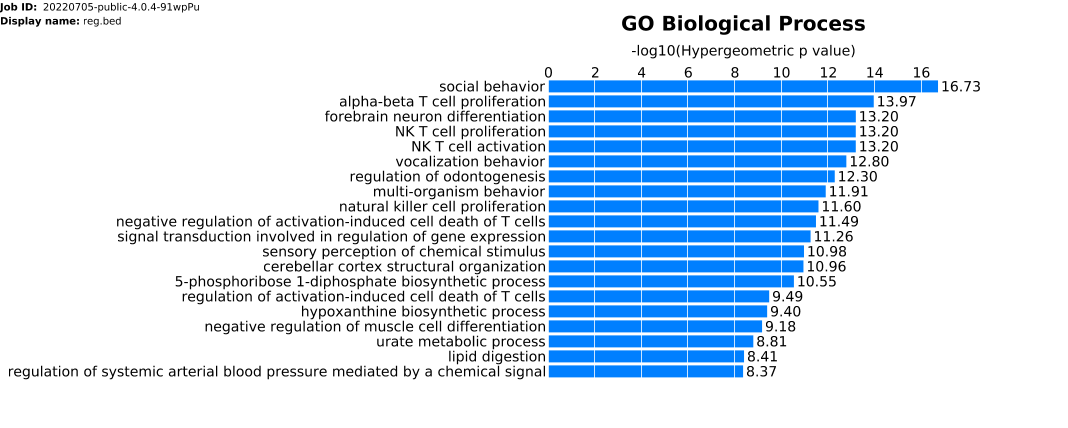
**

**Supplementary Tables**

***Supplementary Table 1: Description of the D-ABS data for low-coverage 4-CpG data simulation***

| **Gene** | **Organism** | **Amplicon Coordinates** | **Genome Assembly** | **Primer FW** | **Primer RV** |
| --- | --- | --- | --- | --- | --- |
| DAO | Human | CHR12:108879926-108880252 | GRCh38/hg38 | aaggTTtgtTTaTaggggTttgaga | ccaActcaaaAAtAcatctAccactc |
| DDOH | Human | CHR6: 110415392-110415789 | GRCh38/hg38 | aTTtaTaaatTagTtggagaaagTTTag | cctattcaAacacactcccaaactcc |
| SCRN1 | Human | CHR7:29990018-29990346 | GRCh38/hg38 | gatatggaatTttggTttagtta | ctttActaAatttttatttctt |
| CDKL5 | Mouse | CHRX: 160994844-160994655 | GRCm38/mm10 | AgAgggTTAgAATAAgAAATTTTggTT | TCTAAAAACAAACTAATAAACACAC |
| DDO_R3 | Mouse | CHR10:40629085-40629513 | GRCm38/mm10 | GTttttTTaTatgtTttggagTTt | acctccctAaaaAtcatttAattcta |
| DDO_R4 | Mouse | CHR10:40629544-40629949 | GRCm38/mm10 | GtgtgtttTtgaggaggtgaTaTtTa | aActtaccctccattAAtccatAcc |
| DDO_R6 | Mouse | CHR10:40630278-40630682 | GRCm38/mm10 | TTtagtgttaaTttattagagTtgtgg | AAtacaatcccttcttAcaacaAAca |
| DDO_R7 | Mouse | CHR10:40630812-40631211 | GRCm38/mm10 | GagggagttgggTatggagTaTaTata | AactctaAAAaAcaAacacaAaAAtc |
| DLX6 | Mouse | CHR6:6864874-6865260 | GRCm38/mm10 | tTataatgTattgttagtgttggaga | tcaAtcctaaAAaaAcaAccaAttcR |
| TPH1a | Zebrafish | CHR25:8159799-8160116 | GRCz11/danRer11 | atttgTtgtTaggaggaagattaag | cacaacatcaaattctctacat |

*(*genes: human_DDO: human D-Aspartate Oxidase, mouse_DDOR4: mouse D-Aspartate Oxidase Region 4, mouse_DDOR6: mouse D-Aspartate Oxidase Region 6, mouse_DDOR7: mouse D-Aspartate Oxidase Region 7*)*

***Supplementary Table 2: Description of the simulated 4-CpGs datasets***


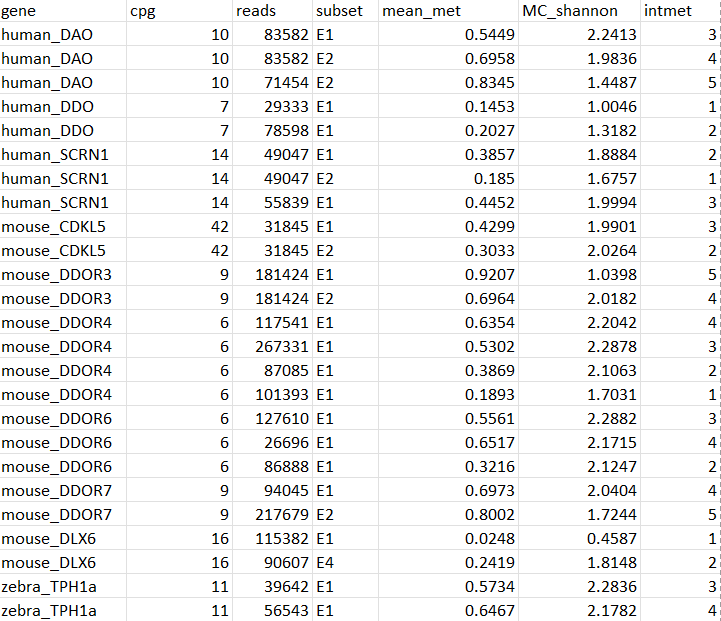


(genes: human_DDO: human D-Aspartate Oxidase, mouse_DDOR4: mouse D-Aspartate Oxidase Region 4, mouse_DDOR6: mouse D-Aspartate Oxidase Region 6, mouse_DDOR7: mouse D-Aspartate Oxidase Region 7. column names: subset: simulated short-read region made up of 4 CpG sites, mean_met: average methylation of the simulated high-coverage short-read dataset, MC_shannon: shannon entropy computed on the high-coverage short-read dataset, intmet: average methylation-based group.)

***Supplementary Table 3: simulated datasets based grouped by average methylation***

| group | number of datasets | average methylation | average coverage |
| --- | --- | --- | --- |
| G1 | 4 | 0-0.2 | 100974.8 |
| G2 | 6 | 0.2-0.4 | 93305.17 |
| G3 | 6 | 0.4-0.6 | 156852.3 |
| G4 | 6 | 0.6-0.8 | 73788.75 |
| G5 | 3 | 0.8-1 | 70678.33 |

***Supplementary Table 4:* Jensen-Shannon distance cutoff as a function of coverage**

| **coverage** | **cutoff** |
| --- | --- |
| 25 | 0.37 |
| 50 | 0.26 |
| 75 | 0.21 |
| 100 | 0.18 |
| 150 | 0.15 |
| 200 | 0.13 |

**Supplementary Table 5: RRBS datasets adopted in the study**

| **Dataset** | **GEO accession** | **Sample accessions** | **Description** |
| --- | --- | --- | --- |
| Dataset 1 | GSE130735 | GSM3752619, GSM3752620,  GSM3752621 | samples from 3 WT littermate E8.5 embryos |
| Dataset 2 | GSE66121 | GSM1614765,  GSM1614766,  GSM1614767 | human CD19+ B-cells isolated from 3 normal controls |
| Dataset 3 | GSE53714 | GSM1299332,  GSM1299333,  GSM1299334 | liver samples from 3 F1 mice originating from C57BL/6J and DBA/2J strain cross |
| Dataset 4 | GSE66121 | GSM1614729,  GSM1614730,  GSM1614731 | human CD19+ B-cells isolated from 3 chronic lymphocytic leukemia |
| Dataset 5 | GSE72700 | GSM1868584,  GSM1868589,  GSM1868591 | ERRBS data from C57BL6 male mice neurons at different developmental stages (hippocampal precursors:, granule cells:; CA3 neurons:) |
